# Overlap syndrome in a 12-year-old girl with systemic lupus erythematosus and anti-oj antibody-positive polymyositis: a case report

**DOI:** 10.1186/s12969-022-00753-z

**Published:** 2022-10-21

**Authors:** Kan-Hsuan Lin, Jun-Kai Kao

**Affiliations:** 1 Frontier Molecular Medical Research Center in Children, Changhua Christian Children Hospital, 135 Nanhsiung Street, Changhua County Changhua, 500 Taiwan; 2 Institute of Biomedical Sciences, National Chung Hsing University, Taichung city, Taiwan; 3 School of Medicine, Kaohsiung Medical University, Kaohsiung city, Taiwan; 4 Department of Post-Baccalaureate Medicine, College of Medicine, National Chung Hsing University, Taichung city, Taiwan

**Keywords:** Overlap Syndrome, Systemic Lupus Erythematosus, Polymyositis, Anti-OJ antibody

## Abstract

**Background:**

The peculiar presentation of overlap syndrome in children makes precise diagnosis difficult. Children with overlap syndrome may or may not have specific antibodies. We present the case of a 12-year-old girl diagnosed with overlap syndrome of systemic lupus erythematosus (SLE) and juvenile polymyositis (JPM) who tested positive for anti-OJ antibodies.

**Case presentation:**

We describe the case of a 12-year-old girl diagnosed with SLE at the age of 7 and presented with fever with malar rash, periungual erythema, generalized weakness, and multiple joint pain at admission. The patient had persistent joint pain and weakness after intravenous methylprednisolone administration and complained of an inability to walk with a positive test for Gower's sign one week after admission, accompanied by elevated alanine aminotransferase (ALT) and creatine-phospho-kinase (CPK) levels. The results of nerve conduction velocity test were normal. Electromyography revealed abundant spontaneous activity and myopathic motor unit action potentials in the right deltoid, biceps, and iliopsoas, in addition to fibrillation and mild myopathic motor unit action potentials in the right rectus femoris muscle. Magnetic resonance imaging revealed diffusely increased signal intensities in the myofascial planes of the bilateral iliopsoas, gluteus, obturator, pectineus, and hamstring muscles. Anti-nuclear antibody, anti-RNP, and rheumatoid factor IgG tests were positive, and inflammatory myopathy autoantibodies revealed anti-OJ antibody positivity, which strongly indicated autoimmune myositis. High-resolution computed tomography of the lung revealed mild pericardial effusion without any evidence of interstitial lung disease. We initiated intravenous pulses of methylprednisolone treatment, followed by cyclosporine, mycophenolate mofetil, and oral steroids. Clinical improvement with a delayed, slowly reduced CPK level after the above treatment and she was discharged after the 18th day of hospitalization.

**Conclusion:**

Overlap syndrome with inflammatory myositis can occur years later in pediatric SLE cases. We should be alert when patients with SLE develop a new presentation characterized by decreased SLE-specific autoantibody titers, positive anti-RNP antibodies, and elevated CPK. Treatment of the overlap syndrome of SLE and JPM is individualized, and the course differs between pediatric and adult patients.

## Background

Connective tissue diseases (CTDs) are inflammatory conditions with characteristic signs and symptoms that define specific disorders. However, some children simultaneously manifest signs and symptoms characteristic of two or more major rheumatic disorders, such as juvenile idiopathic arthritis, SLE, juvenile dermatomyositis, cutaneous systemic scleroderma, and vasculopathy. Children with these disorders are often difficult to categorize according to the existing classification criteria and are referred to as overlap syndromes. Children with overlap syndrome may or may not have specific antibodies, and cannot be assigned to a single disease entity. Sometimes, the presentation is so peculiar that a precise diagnosis is clinically very difficult, and specific treatment is not initially possible. Additionally, fatal outcomes may occur before a diagnosis.

Here, we report a case of SLE diagnosed at the age of 7 years, with fever and skin rash as the initial presentation. Raynaud's phenomenon and joint pain developed during four years of treatment. At the age of 12 years, she presented with fever, malar rash, periungual erythema, multiple joint pain, and muscle weakness, which eventually led to the diagnosis of overlap syndrome of SLE and JPM with positive anti-OJ antibodies. This case report was approved by the institutional review board of Changhua Christian Hospital (IRB number 220113).

## Case report

A 12-year-old girl was admitted to Changhua Christian Children’s Hospital in May 2021, complaining of intermittent low-grade fever for one week and SLE flare-up. This girl was diagnosed with SLE in 2017 and presented with low-grade fever, malar-distributed facial rash, oral ulcers, and enlarged lymph nodes. The examination revealed a hemoglobin level of 10.4 g/dL and a platelet count of 2.88 × 10^5^ mm^3^. Urinalysis revealed proteinuria but no hematuria. The liver function test showed an ALT level of 22 U/L; however, the renal function test results were normal. There were strong positive antinuclear antibodies (ANA) (1:1280 pattern), anti-dsDNA (684 IU/mL), positive anti-sm, positive anti-SSA, and anti-RNP(2.0 AI); C3 and C4 levels were 31.6 & below 6.7 (mg/dL, respectively).

She was initially administered pulse corticosteroid therapy and then treated with mycophenolate mofetil, azathioprine, and hydroxychloroquine. During the four years of treatment, she had an uneven clinical course and persistently low complement levels. Two years after being diagnosed with SLE, Raynaud's phenomenon affecting the fingers was observed, along with new-onset wrist and knee joint pain; therefore, she received additional methotrexate. One week before hospitalization, she developed fever and was treated with Baktar in the oral form (sulfamethoxazole 400 mg + trimethoprim 80 mg) for a suspected urinary tract infection. However, the fever persisted and was accompanied by an obvious malar rash, periungual erythema, generalized weakness, and multiple joint pains (wrist, knee, and fingers without swelling or limited range of motion). The patient was then admitted for further evaluation and treatment. Physical examination revealed ulcers on the bilateral buccal surfaces; rash on the face (malar distribution, presented with erythema over cheeks; nasal bridge but with spared nasolabial folds); enlarged right cervical lymph node with tenderness; right flank knocking pain; and localized heat on the right knee. Erythematous vasculitic purpuric plaques are present on the palmar surfaces and soles with some abrasion. The fingertips turned red without ulceration or gangrene but with periungual telangiectasias. Physical examination did not reveal skin lesions such as Gottron’s papules, heliotrope eruption, shawl sign, Holster sign, or calcinosis.

We initially prescribed intravenous methylprednisolone (2 mg/kg/day). Although the fever subsided, the malar rash faded, and the vasculitis in her fingers improved, she complained of joint pain and weakness. Furthermore, the liver enzyme levels had been continuously elevated (Table 1). One morning, after one week of admission, she complained of an inability to walk and demonstrated a positive test for Gower's sign, which indicates weakness of the proximal muscles.

**Table 1:** The results of blood chemistry, immunology, and serology tests at time of SLE and overlap syndrome being diagnosed

	SLE	Overlap syndrome
C3/C4	31.6/ < 6.7 mg/dL	63.6/15.9 mg/dL
ANA	1:1280	1:320
dsDNA	Strong positive 684.0 IU/mL	Equivocal 11.93 IU/mL
Anti-sm	Positive > 8.0 AI (Negative < 1.0)	Negative 2.53 EliA U/mL (Negative < 7.0)
Anti-SSA	Positive 2.0 AI	Negative 1.63 EliA U/mL
Anti-SSB	Negative < 0.2 AI	Negative < 0.3 EliA U/mL
Anti-cardiolipin IgG	Negative 9.1 GPL-U/mL	Negative 1.72 GPL-U/mL
Anti-cardiolipin IgM	Negative 1.5 MPL-U/mL	Negative 3.2 MPL-U/mL
Anti-β2 glycoprotein I IgG	Negative 11.9 U/mL	–
RNP	Positive 2.0 AI (Negative < 1.0)	Positive 14.5 EliA U/mL (Negative < 5.0)
ALT	22 U/L	1154 U/L
CPK	–	3517 U/L
Myoglobin (Urine)	–	3734 μg/L
Proteinuria	196.3 mg/day	495.1 mg/day
ESR	50 mm/hr	14 mm/hr
CRP	0.12 mg/dL	< 0.02 mg/dL

**Fig. 1:**
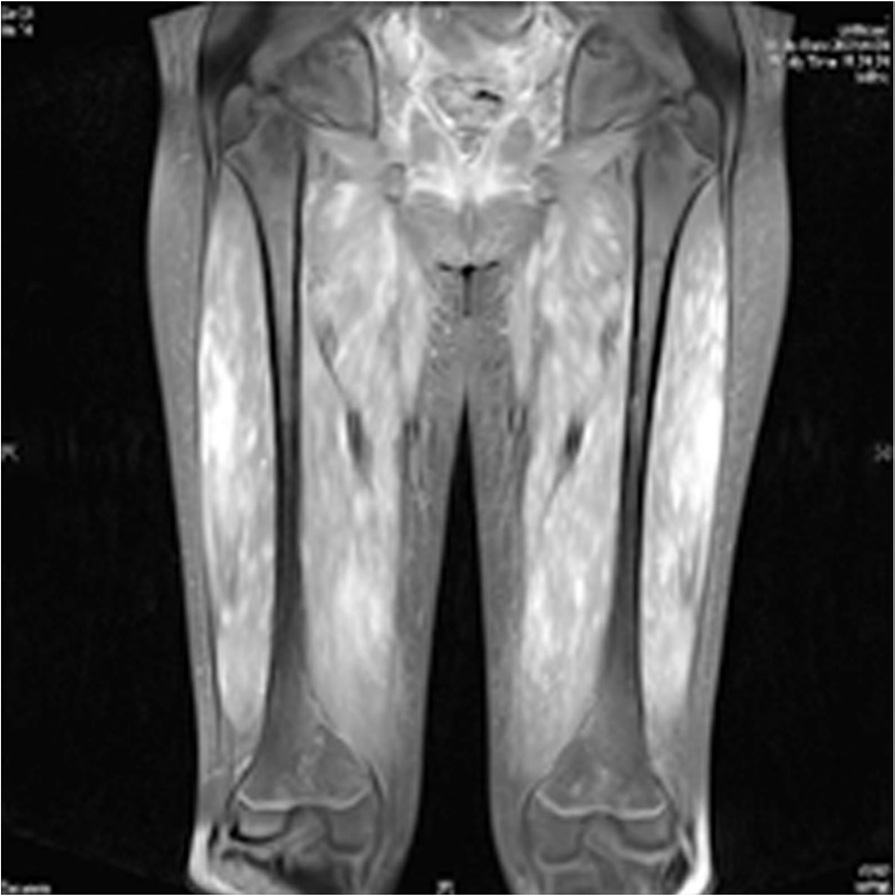
MRI with gadolinium-DTPA enhancement revealed diffusely increased signal intensities in the myofascial planes of the bilateral iliopsoas, gluteus, obturator, pectineus, and hamstring muscles in the proton density image

On investigation, the hemoglobin was 13.5 g/dL and the platelet count was 2.77 × 10^5^ mm^3^. Urinalysis revealed proteinuria but no hematuria; results of renal function tests were normal. However, the ALT level increased to 1076 U/L. Serum CPK levels were elevated (3517 U/L). Based on this clinical picture and the laboratory results, autoimmune inflammatory muscle disease was suspected. The nerve conduction velocity test (NCV), electromyography (EMG), and magnetic resonance imaging (*MRI)* were performed. The results showed normal NCV. EMG revealed abundant spontaneous activity and myopathic motor unit action potentials in the right deltoid, biceps, and iliopsoas, in addition to fibrillation and mild myopathic motor unit action potentials in the right rectus femoris. Magnetic resonance imaging (MRI) with gadolinium-DTPA enhancement revealed diffusely increased signal intensities in the myofascial planes of the bilateral iliopsoas, gluteus, obturator, pectineus, and hamstring muscles (Fig. 1). Echocardiography and abdominal ultrasonography were performed. Echocardiography revealed normal left ventricular function, without pulmonary arterial hypertension. Abdominal ultrasonography revealed a normal liver size without dilatation of the biliary tract, a normal portal vein, and no ascites. The likely normal echogenicity helped prove that high ALT level was caused by myositis and not by hepatic inflammation.

**Fig. 2:**
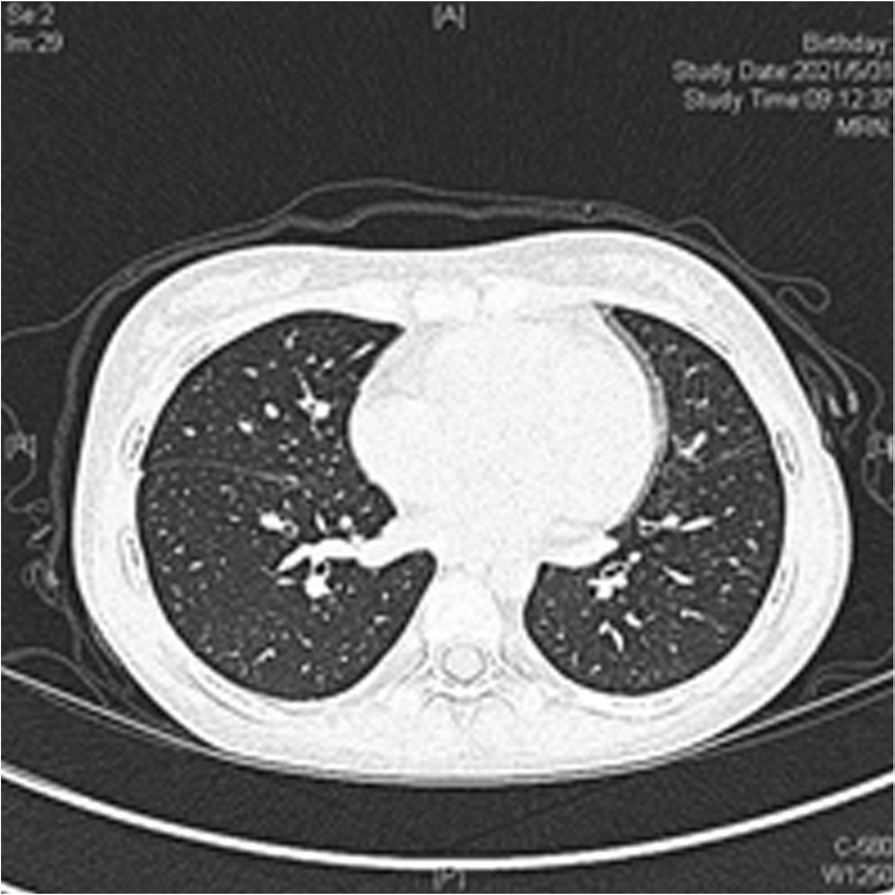
Chest high-resolution computed tomography (HRCT) images reveal thickening of the pericardium with mild pericardial effusion. No definite evidence of pulmonary nodules and associated pleural effusion. Neither bronchiectasis nor areas of air-trapping was showed

Serological examination revealed ANA (1:320 pattern), anti-RNP(14.5 EliA U/mL), and RF IgG positivity. However, the anti-Sm and anti-SSA/SSB antibodies were negative (Table 1). The anti-dsDNA antibody was equivocal (11.93 IU/mL), while the inflammatory myopathy autoantibodies were anti-OJ positive (Table 2). Since the MRI results and positive inflammatory myopathy-specific antibodies strongly indicated autoimmune myositis, overlap syndrome was diagnosed without a muscle biopsy. Pulmonary function should be evaluated in patients with anti-OJ autoantibodies because of the high incidence of ILD. However, considering the transmission risk during the pandemic COVID -19, we performed high-resolution computed tomography (HRCT) of the lungs instead of single-breath diffusion capacity. The results showed thickening of the pericardium with mild pericardial effusion. No definite evidence of pulmonary nodules or associated pleural effusions was found. No bronchiectasis or air trapping areas were observed. (Fig. 2).

**Table 2:** The results of inflammatory myopathy autoantibodies test

**Myositis-specific autoantibodies (MSAs)**	
Mi-2α	Negative
Mi-2β	Negative
TIF1γ	Negative
MDA5	Negative
NXP2	Negative
SAE	Borderline
Jo-1	Negative
SRP	Negative
PL-7	Negative
PL-12	Negative
EJ	Negative
OJ	Positive
**Myositis-associated autoantibodies (MAAs)**	
Ku	Negative
PM-Scl 100	Negative
PM-Scl 75	Negative
Ro-52	Negative

**Fig. 3:**
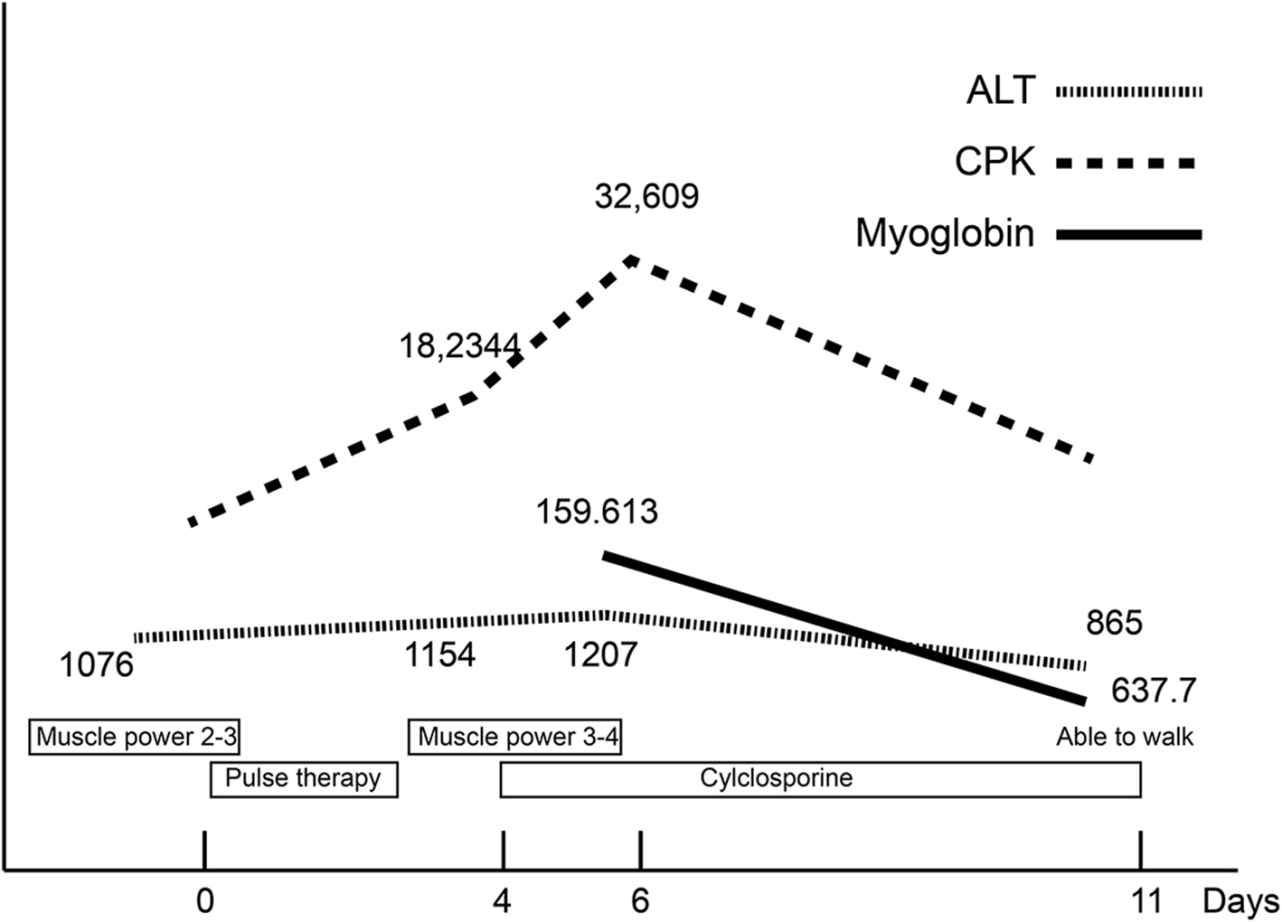
Chart of clinical course, treatment and laboratory results

We started treatment with intravenous pulses (11 mg/kg/day) of methylprednisolone for three consecutive days. Two days later, she received cyclosporine due to persistently elevated CPK levels. Mycophenolate mofetil (57 mg/kg/day) and oral steroids (1.2 mg/kg/day) were added to the management. After the above treatment, clinical improvement occurred with a relatively delayed and slow reduction in CPK levels (Fig. 3). The patient was discharged after the 18th day of hospitalization with CPK 13,354 U/L (Fig. 3).

## Discussion

Overlap syndrome is defined as the coexistence of signs, symptoms, and immunological features of two or more connective tissue diseases that occur simultaneously, sequentially, or at different times in the same patient [1]. Although this concept appears uncomplicated, this rare disease is difficult to diagnose because its genetics and pathogenesis are not completely understood. In the overlap syndrome, mixed connective tissue disease (MCTD) is now treated as a well-defined entity with clearer diagnostic criteria.

Our case fulfilled the diagnostic criteria for MCTD according to the Kasukawa diagnostic criteria. However, we still do not think the patient is a case of MCTD. The main reason is that, nowadays, MCTD is recognized as a distinct entity with unique clinical features, therapeutic response, and prognosis. MCTD is characterized by the concomitant occurrence of clinical symptoms of different rheumatic disorders without meeting the clear diagnostic criteria for these diseases. Our patient met the diagnostic criteria for both SLE and polymyositis. Besides the low positive anti-RNP titer (two-fold ratio related to negative values), the patient had strong anti-dsDNA, anti-Sm, and myositis-specific antibodies, which are rare in MCTD. These results convinced us that the patient had overlap syndrome rather than MCTD [2, 3].

The descriptions of pediatric overlap syndromes are limited to a few case reports and case series [4–6]. In pediatric patients initially diagnosed with SLE, the heterogeneity of presentation and course makes the diagnosis of overlap syndrome even more difficult. In clinical practice, the overlap between SLE and systemic sclerosis is more common in East and South Asian populations [1]. Our patient was diagnosed with overlap syndrome with SLE and JPM. The prevalence of overlap between SLE and idiopathic myositis has been reported to be 3.4–6.3% [7, 8]. In a study of adult and pediatric patients with SLE by Bitencourt et al*.*, 6.3% of the patients developed inflammatory myositis. A significantly higher prevalence of overlap myositis was found in childhood SLE patients than in adult patients [9].

At the time of SLE diagnosis, anti-RNP autoantibodies were detected in our patient. Previous reports have shown that the overall disease development in SLE-myositis patients is influenced by the presence of anti-RNP autoantibodies [1, 7, 9]. Therefore, SLE patients with anti-RNP autoantibodies must be monitored meticulously at clinical presentation or follow-up for overlap myositis [1, 9]. Interestingly, as the muscle discomfort and joint symptoms of our patient worsened, her SLE-specific autoantibody levels declined (Table 1). This may warn the clinicians that a patient has developed an overlap.

Our patient developed Raynaud's phenomenon and fingertip vasculitis two years after the initial diagnosis and developed overlap syndrome with SLE and JPM four years after the SLE diagnosis. The overlap syndromes could be diagnosed concurrently with SLE or developed after SLE diagnosis [1, 7, 9], and inflammatory myositis occurred at an average of 5.25 years after SLE diagnosis [9]. In a juvenile case report by Nitta et al*.*, a 16-year-old girl presented with overlap syndrome consisting of SLE from the age of 7 years and JPM from the age of 10, which was later accompanied by systemic sclerosis from the age of 15. However, in this case, Raynaud's phenomenon and pitting ulcers of the fingers and toes occurred one year after the diagnosis of SLE-JPM overlap syndrome, which is different from our case [10].

In contrast to anti-Mi-2 or anti-Jo-1 antibodies, which are more common in autoimmune myositis, our patient tested positive for anti-OJ autoantibodies. Anti-OJ autoantibodies are myositis-specific autoantibodies that target isoleucyl-tRNA synthetase and are associated with anti-synthetase syndrome (ASS) [11]. Anti-OJ autoantibodies can only be found in less than 5% of patients with idiopathic inflammatory myopathies [12]. In addition to arthritis, fever, and Raynaud phenomenon, which occur in ASS, patients with anti-OJ antibodies appear to have more severe myositis. In the study by Noguchi et al*.*, an increased incidence of severe limb and neck muscle weakness, dysphagia, and muscle atrophy was noted in muscle biopsies [11, 13]. Skin involvement may occur in patients who are anti-OJ positive. Patients have been reported to have heliotrope rash, Gottron's sign or papules, V sign, shawl sign, and holster sign. Compared to adult cases, ASS is rare in juvenile disease and seems to cause a lower incidence of ILD in pediatric cases [11–13]. Considering the transmission risk during the pandemic COVID-19, a pulmonary function test, especially the single-breath diffusion capacity test, was not performed in our case. The unremarkable HRCT findings in our case imply that there may be differences in the clinical features of positive anti-OJ antibodies between juvenile and adult cases [14].

The gold standard to characterize idiopathic inflammatory myopathies is the morphological, immunohistochemical and immunopathological analysis of muscle biopsy. We did not perform muscle biopsy for three reasons. First, the clinical presentation and the result of MRI revealed that JPM/JDM was highly suspected. Second, autoantibody testing is an important tool for the diagnosis of IIMs. Myositis‑specific autoantibodies such as anti-OJ antibody are almost exclusively present in IIMs. The last reason we did not perform muscle biopsy is that her parents hesitated to do the examination. In fact, a muscle biopsy is not only to confirm IIMs but also helpful to identify the subset of IIMs [15, 16].

Most patients with anti-OJ have a good response to glucocorticoids [11]. However, patient treatment should be individualized according to different manifestations [17, 18]. Management guidelines are based on clinical manifestations and individual profiles. Therapy for the overlap syndrome usually requires a cocktail of drugs to suppress inflammation. Myositis manifesting clinically as muscle weakness is more common than the laboratory elevation of muscle enzymes. The response to treatment should be mainly evaluated by clinical improvement because, as in our patient, CPK levels do not always correlate with disease activity [19].

## Conclusion

Although rare, overlap syndrome with inflammatory myositis can occur over the years in pediatric SLE cases. Physicians should be alert while treating patients with SLE who develop a new apparent rheumatic presentation with a decreased SLE-specific autoantibody titer, positive anti-RNP antibodies, and elevated CPK. The treatment for overlap syndrome with SLE and JPM is individualized, and the progress differs between pediatric and adult patients.

## Data Availability

Data sharing is not applicable to this article as no datasets were generated or analysed during the current study.

## Abbreviations

SLE: Systemic lupus erythematosus
JPM: Juvenile polymyositis
MCTD: Mixed connective tissue disease
ANA: Anti-nuclear antibody
EMG: Electromyography
MRI: Magnetic resonance image
RF: Rheumatoid factor
ILD: Interstitial lung disease
HRCT: High-resolution computed tomography
ASS: Anti-synthetase syndrome
